# Real-Life Insights into Pertussis Diagnosis: High Yield of PCR Testing and Clinical Outcomes—An Emerging Old Enemy or Just a Sign of PCR Times?

**DOI:** 10.3390/jpm14121116

**Published:** 2024-11-22

**Authors:** Ilias E. Dimeas, Ourania S. Kotsiou, Polyxeni Salgkami, Irene Poulakida, Stylianos Boutlas, Zoe Daniil, Georgia Papadamou, Konstantinos I. Gourgoulianis

**Affiliations:** 1 Department of Respiratory Medicine, Faculty of Medicine, University of Thessaly, Biopolis, 41500 Larissa, Greece; okotsiou@uth.gr (O.S.K.); xsalgami@gmail.com (P.S.); zdaniil@uth.gr (Z.D.); kgourg@uth.gr (K.I.G.); 2 Laboratory of Human Pathophysiology, Department of Nursing, University of Thessaly, Gaiopolis, 41110 Larissa, Greece; 3 Department of Emergency Medicine, Faculty of Medicine, University of Thessaly, Biopolis, 41500 Larissa, Greece; irene.poulakida@yahoo.com (I.P.); sboutlas@gmail.com (S.B.); georgia.papadamou@yahoo.com (G.P.)

**Keywords:** *Bordetella pertussis*, community acquired pneumonia, lower respiratory tract infection, PCR testing, whooping cough

## Abstract

**Background/Objectives:** Pertussis remains a significant public health concern despite effective vaccines due to diagnostic challenges and symptom overlap with other respiratory infections. This study assesses the prevalence of *Bordetella pertussis* using advanced polymerase chain reaction (PCR) testing and examines the clinical outcomes over a one-month follow-up. **Methods:** We conducted a cross-sectional study at the University Hospital of Larissa, Greece, from April to June 2024, collecting 532 nasopharyngeal swabs from patients with respiratory symptoms. Diagnostic testing utilized the BioFire^®^ Respiratory 2.1 Plus Panel. Demographics, clinical presentations, vaccination histories, and clinical outcomes were systematically recorded and analyzed. **Results:** Of 532 patients, 47 (8.8%) were diagnosed with pertussis. The mean age was 61.87 ± 13.4 years; 57.4% were female. Only 12.8% had contact with known pertussis patients. Regarding vaccination history, 36.2% had received diphtheria, tetanus, and pertussis vaccines, with the last dose administered an average of 46 years prior to this study. The primary symptom was cough (100%), with additional symptoms including fever (36.2%) and paroxysmal cough (34%). Six patients (12.8%) required hospitalization due to pneumonia and severe respiratory failure. All patients received successful treatment; however, 23.4% reported persistent post-infectious cough at the one-month follow-up. **Conclusions:** PCR testing significantly improved the diagnosis of pertussis among adults presenting with respiratory symptoms. The findings highlight the need for updated vaccination strategies and improved diagnostic protocols to effectively manage pertussis and reduce its public health impact.

## 1. Introduction

Pertussis, commonly known as whooping cough, is a highly contagious respiratory disease caused by the bacterium *Bordetella pertussis*. Despite the widespread availability of effective vaccines, pertussis continues to pose a significant public health challenge globally, with increasing incidence rates reported in both low- and high-income countries [1]. This resurgence is not limited to areas with incomplete vaccination coverage but also extends to countries with well-established immunization programs, highlighting the complexity of controlling this infection [2].

In 2022, a total of 2623 pertussis cases were documented across 29 countries within the European Union (EU) and the European Economic Area (EEA) [2]. Notably, Germany and Poland accounted for 60% of these reported cases, indicating a concentrated burden in these nations [2]. The overall notification rate for pertussis in 2022 was 0.7 cases per 100,000 individuals, representing a slight increase compared to 2021 [2]. This uptick follows a significant reduction in pertussis cases observed in 2019 and during the COVID-19 pandemic in 2020, periods marked by enhanced public health interventions and social distancing measures that inadvertently curtailed the transmission of various respiratory pathogens [2]. Infants under one year of age remained the most vulnerable group, exhibiting the highest notification rate of 4.0 per 100,000 population [2]. Additionally, individuals aged 15 years and older constituted 70% of all reported pertussis cases, underscoring a shift in the epidemiological landscape of the disease [2].

Recent epidemiological studies have highlighted a shift in the burden of pertussis towards older children and adults [3,4,5,6,7,8,9]. This shift is attributed to several factors, including waning immunity from both natural infection and vaccination, detection bias introduced by improved diagnostic technologies, and changes in vaccine formulations. Specifically, the global shift from whole-cell pertussis vaccines (wP) to acellular pertussis vaccines (aP), which, although associated with fewer side effects, are known to provide shorter-lasting immunity. This change in vaccine formulation, implemented in many high-income countries during the late 1990s and early 2000s, has contributed to the increasing incidence of pertussis in older children, adolescents, and adults, underscoring the need for booster vaccinations [7,8,9].

Despite these advancements, the true burden of pertussis in adults is likely underestimated due to widespread under-recognition by healthcare professionals (HCPs), underdiagnosis, and under-reporting in this age group [7,8,9]. Non-standardized testing guidance and varied case definitions have further contributed to the under-reporting of pertussis cases [7,8,9]. Traditional diagnostic techniques, including bacterial culture and serological testing, have significant drawbacks [3]. While bacterial culture is considered the gold standard for diagnosis, it is time-consuming and may produce false-negative results, especially if antibiotic treatment has already begun [3]. Serological tests, on the other hand, are less effective in identifying acute infections and struggle to differentiate between recent and past infections, limiting their usefulness in timely diagnosis [3,4,5,6,7,8,9].

Moreover, despite the limitations of traditional diagnostic methods, the similarity of its symptoms with other respiratory infections leads to underdiagnosis [3,4,5,6,7,8,9]. Pertussis often presents with symptoms that closely resemble those of other upper respiratory tract infections (URTIs), such as a severe cough and vomiting after coughing (post-tussive emesis). This symptom overlap complicates the early and accurate diagnosis of the disease [3,4,5,6,7,8,9]. Underdiagnosis of *Bordetella pertussis* often leads to unnecessary antibiotic prescriptions for other URTIs, contributing to the growing problem of antimicrobial resistance [5,6,7,8,9].

The introduction of polymerase chain reaction (PCR) testing has significantly improved the diagnostic accuracy for *Bordetella pertussis* [4]. PCR assays detect *Bordetella pertussis* DNA with high sensitivity and specificity, even during the early stages of infection and from various sample types, such as nasopharyngeal swabs [4,5,6,7,8,9]. This advanced technique not only allows for earlier and more reliable diagnosis but also helps identify cases that might be missed by traditional methods [4,5,6,7,8,9]. Enhanced diagnostic accuracy through PCR can reduce misdiagnosis and the consequent inappropriate use of antibiotics, which is crucial given the rising concern of antibiotic resistance [5,6,7,8,9].

In this context, a screening program conducted at the beginning of 2024 in the Emergency Department (ED) of the University Hospital of Larissa in Central Greece revealed an unexpectedly high rate of pertussis cases within our hospital’s service area. This finding prompted the implementation of a monitoring protocol aimed at assessing clinical data and outcomes in these patients to gain a deeper understanding of their clinical trajectories. By leveraging advanced diagnostic tools like PCR, our study seeks to provide real-life insights into the prevalence of pertussis and its clinical outcomes, thereby addressing the ongoing challenges of underdiagnosis and informing more effective public health strategies.

## 2. Materials and Methods

### 2.1. Study Design and Setting

This observational cross-sectional study was conducted at the University Hospital of Larissa in Central Greece from April to June 2024. Serving as the primary healthcare facility for the entire Thessaly region—Greece’s third most populous area, home to 687,527 people, with over 230,000 residents in the metropolitan area of Larissa—the University Hospital of Larissa is ideally positioned to provide comprehensive data on respiratory tract infections (RTIs) within a large and diverse population.

### 2.2. Inclusion and Exclusion Criteria

This study included adult patients who presented with symptoms indicative of RTIs, such as persistent cough and fever, at participating health centers within the Thessaly region. Eligibility was further determined by the availability of a nasopharyngeal swab sample collected during the study period from April to June 2024. Additionally, participants were required to provide informed consent for their participation and the use of their data in the study. Conversely, individuals were excluded if their clinical or epidemiological data were incomplete, if they were under 18 years of age (based on the study’s focus), or if they declined to participate or provide consent. These criteria ensured that the study population was well-defined and that the data collected were comprehensive and reliable for analysis. Selection bias was minimized by including all eligible patients during the study period, ensuring a representative sample.

### 2.3. Sample Collection and Diagnostic Testing

Nasopharyngeal swabs were collected from patients appearing with respiratory symptoms, including persistent cough, fever, and respiratory distress. To ensure comprehensive pathogen detection, the BioFire^®^ Respiratory 2.1 Plus Panel (BioFireDx, Marcy-l’Étoile, France) was employed.

This multiplex polymerase chain reaction (PCR) panel is capable of identifying a broad spectrum of upper respiratory pathogens, including *Bordetella pertussis*. Specifically, the panel tests for the following viruses: Adenovirus, Coronaviruses (229E, HKU1, NL63, OC43), Middle East Respiratory Syndrome coronavirus (MERS-CoV), Severe Acute Respiratory Syndrome coronavirus 2 (SARS-CoV-2), Human Metapneumovirus, Human Rhinovirus/Enterovirus, Influenza A and B, Parainfluenza viruses (1–4), and Respiratory Syncytial Virus (RSV), as well as the following bacteria: Chlamydia pneumoniae, Mycoplasma pneumoniae, and Bordetella parapertussis. The selection of the BioFire^®^ Respiratory 2.1 Plus Panel was based on its high sensitivity and specificity, rapid turnaround time, and ability to simultaneously detect multiple pathogens, thereby facilitating a comprehensive diagnostic approach [8].

### 2.4. Data Collection

Patient epidemiological charts were meticulously reviewed to extract a comprehensive range of sociodemographic and clinical data. The collected variables included sociodemographic information, such as age, gender, place of origin, and recent travel or immigration history. Additionally, exposure history was documented, specifically noting any contact with known pertussis cases. Vaccination status was assessed by recording the type of vaccine received (DTP or DTaP) and the timing of the last vaccination dose. Clinical data encompassed the duration from symptom onset to diagnosis, symptom profile (including severity and type of cough, presence of fever, and post-tussive emesis), and prior antibiotic use. Outcomes were evaluated by determining the necessity for hospital admission, the occurrence of severe complications (e.g., pneumonia, respiratory failure), and overall clinical outcomes. Furthermore, follow-up data were collected to assess residual symptoms one month post-diagnosis, with a particular focus on persistent cough. A comprehensive database was established to systematically organize and store the collected data, facilitating efficient analysis and ensuring data integrity.

### 2.5. Ethical Considerations

The study was conducted in accordance with the Declaration of Helsinki and local ethical guidelines to protect participants’ rights and well-being. Ethical approval was obtained from the Human Research Ethics Committee of the University Hospital of Larissa (protocol code: 8/17th and date of approval: 25 October 2023). Informed consent was secured from all participants.

### 2.6. Statistical Analysis

Data analysis was performed using IBM SPSS Statistics (version 25, Armonk, NY, USA), utilizing both descriptive and inferential methods to identify relationships and trends within the dataset. Continuous variables were summarized as means with standard deviations (±SD) for normally distributed data and as medians with standard errors (±SE) for non-normal data. Categorical variables were presented as frequencies and percentages. The Kolmogorov–Smirnov test assessed normality, guiding the choice of statistical tests. Normally distributed continuous variables were analyzed using Student’s t-test or one-way ANOVA, while non-normally distributed variables were examined with Mann–Whitney U, Wilcoxon signed-rank, or Kruskal–Wallis tests. Categorical variables were analyzed using Chi-square tests, with Fisher’s Exact Test applied when expected frequencies were low. Multivariate techniques, such as logistic regression, were used to identify independent predictors of pertussis diagnosis and clinical outcomes, adjusting for confounders like age, vaccination status, and comorbidities. A *p*-value of <0.05 was considered statistically significant.

## 3. Results

A total of 532 nasopharyngeal samples from patients presenting with respiratory symptoms were analyzed. The demographic and clinical characteristics of the study population are presented in Table 1. Among these, 47 cases (8.8%) of *Bordetella pertussis* were identified. The mean age of patients was 61.9 ± 13.4 years, with a higher prevalence of females (57.4%, *n* = 27 vs. 42.4%, *n* = 20; *p* = 0.08) compared to males. Contact with a known pertussis case was reported in only 12.8% of patients (*n* = 6), including contacts with children, partners, or colleagues. Regarding vaccination history, 36.2% of patients had received Diphtheria–Tetanus–Pertussis (DTP or DTaP) vaccines, with the last dose administered an average of 46 ± 14.5 years ago.

Clinical presentations varied, with most patients seeking medical attention at the ED within the first 5 ± 2 days of symptom onset. The primary presenting symptom was cough, reported in all 47 cases (100%). Low-grade fever was observed in 36.2% of patients (*n* = 17), and 34% (*n* = 16) exhibited a paroxysmal cough. Other symptoms included sore throat (*n* = 10), headache (*n* = 6), wheezing (*n* = 8), dyspnea (*n* = 6), sneezing (*n* = 4), and rhinorrhea (*n* = 4). Prior to diagnosis, 40.4% of patients (*n* = 19) were empirically treated with fluoroquinolones, while 53.2% (*n* = 25) received β-lactams.

Hospitalization was required for 6 of the 47 patients (12.8%), with stays ranging from 5 to 12 days. Two of these patients developed complications, including pneumonia. Notably, one patient experienced severe respiratory failure necessitating invasive mechanical ventilation and transfer to the intensive care unit (ICU). All hospitalized patients received antibiotic treatment, primarily azithromycin in combination with a β-lactam antibiotic; additional therapies, such as corticosteroids and/or oxygen therapy, were administered based on clinical needs.

Statistical analysis revealed that fever (r = 0.74, *p* < 0.001), whooping cough (r = 0.81, *p* < 0.001), and sore throat (r = 0.37, *p* < 0.001) were significantly associated with hospitalization. All 47 cases were successfully treated, leading to clinical recovery. At the one-month follow-up, none of the patients who were managed in the ED required further hospitalization. However, 23.4% of patients (*n* = 11) reported persistent post-infectious cough.

## 4. Discussion

This study aimed to investigate the occurrence and clinical presentation of *Bordetella pertussis* infection in adult patients presenting with symptoms of respiratory tract infections (RTIs) in a large tertiary hospital in Central Greece. Our findings indicate that despite the widespread availability of vaccines, pertussis remains a relevant clinical entity among adults, posing diagnostic and therapeutic challenges, as 8.8% of the 532 patients tested positive for pertussis, with a mean age of 61.9 years and a predominance of female patients (57%).

The prevalence rate of 8.8% in our study aligns with recent findings from other regions. For instance, a study conducted in Ankara, Turkey, by Ilbay et al. (2022) reported a pertussis prevalence of 3.5% among 115 adults presenting with acute cough, highlighting the under-recognition of pertussis in this demographic [10]. Other studies in Europe, such as those conducted in Germany and Italy, have demonstrated pertussis prevalence rates ranging from 4% to 6% among adults presenting with respiratory symptoms [11,12]. The discrepancy in pertussis prevalence rates across studies can be attributed to several factors, including differences in study design, diagnostic methods, population demographics, and regional vaccination practices.

Similarly, the results from a previous Greek study by our team indicated a low prevalence of anti-PT IgG (>50 IU/mL) in the adult population [9]. Reported pertussis prevalence rates varied considerably, ranging from 0% in the region of the Ionian Islands and West Macedonia to 16.4%, generally reported in the region of Peloponnese [9].

Conversely, higher pertussis prevalence rates have been reported in certain studies. For example, a study conducted in the USA [13] found a prevalence of 12% among adults with prolonged cough. Although adult prevalence is not typically as high, surveillance indicates substantial regional variation. This may reflect the ongoing circulation of pertussis in regions with lower vaccination uptake or waning immunity in older populations [13]. Overall, the prevalence reported in our study aligns more closely with the higher end of the global spectrum, emphasizing the importance of heightened clinical suspicion and targeted prevention strategies. Higher rates [13] may suggest under-recognition of pertussis in regions with waning immunity or differences in case definitions. These variations highlight the need for standardized diagnostic criteria and broader awareness of pertussis in adults.

The mean age of 61.9 years is consistent with other studies indicating that pertussis can impact older populations, who may have waned immunity from childhood vaccinations or have not received recent booster doses [6]. The observed shift towards older age groups in pertussis cases within high-income countries is significantly influenced by waning immunity due to both natural infections and acellular vaccines. Studies indicate that individuals vaccinated during childhood experience a gradual decline in immunity, making them more susceptible to pertussis as they age. For instance, research has shown that older adults often present atypical symptoms of pertussis, leading to diagnostic challenges and under-reporting of cases in this demographic [14]. A retrospective study in England found an incidence of pertussis at 5.8 per 100,000 person-years in adults aged 50 and older, emphasizing the heightened risk of severe complications among older adults [15]. This trend is compounded by the impact of the COVID-19 pandemic, which has altered healthcare-seeking behaviors and potentially hindered timely diagnosis and treatment of pertussis [16]. Given these dynamics, there is an urgent need for public health initiatives to enhance vaccination strategies, including routine booster shots for older populations, to mitigate the rising burden of pertussis among adults [14,16]. The mean age of 61.9 years in our study further emphasizes the vulnerability of older adults, a group that has historically been underdiagnosed and undertreated for pertussis. This is consistent with findings by Cherry et al. (2012), who highlighted the increasing burden of pertussis in adults due to diminished vaccine-induced immunity over time [17].

The predominance of females in our cohort, with 57% of patients being women, aligns with findings from various studies that indicate a higher prevalence of pertussis among females [7]. This trend could potentially be attributed to demographic characteristics within our patient population, as well as factors influencing the susceptibility and reporting of pertussis in different genders [18]. For example, a study conducted in Australia observed that 66% of pertussis cases occurred in women aged 50 and older, suggesting a possible pattern of higher incidence in females across age groups [19]. Additionally, a meta-analysis covering multiple countries indicated that the incidence of pertussis in females was notably higher in certain age categories, particularly among infants and young children [18]. The disparity in pertussis prevalence between genders may be reflective of underlying factors such as differences in healthcare-seeking behaviors, access to healthcare services, and the likelihood of receiving vaccinations. As females often serve as primary caregivers, they may be more exposed to pertussis transmission, particularly from children who are often more symptomatic. Therefore, the higher prevalence of pertussis in females in our cohort could not only reflect actual incidence rates but also demographic patterns within our patient population [18,19]. This suggests that further research is warranted to explore these trends and their implications for public health strategies, particularly in ensuring that vaccination efforts target at-risk populations effectively.

The low rate of known contact with pertussis cases (13%) highlights the difficulty in identifying and managing pertussis outbreaks. This finding aligns with literature suggesting that pertussis transmission often occurs in close contact settings, and asymptomatic or mild cases can contribute to missed diagnoses. Improving surveillance and contact tracing is crucial to better manage outbreaks and prevent further spread [9,20,21].

Our study found that 38% of pertussis-positive patients had received DTP or DTaP vaccines, with the last dose administered an average of 46 years ago. This long interval since the last vaccination likely contributed to the susceptibility observed in older adults. Previous research supports this, demonstrating that immunity wanes significantly within two decades post-vaccination, thereby increasing the risk of pertussis infection in older populations [22]. Furthermore, the transition from whole-cell to acellular vaccines has been associated with a more rapid decline in immunity, which may partially explain the high prevalence of pertussis in our adult cohort [4].

The high diagnostic yield of the BioFire^®^ Respiratory 2.1 Plus Panel, which detected *Bordetella pertussis* among other pathogens, illustrates the significant advantages of advanced molecular diagnostics for detecting *Bordetella pertussis* among other pathogens. PCR-based testing enhances diagnostic accuracy and helps distinguish pertussis from other respiratory infections, ultimately reducing unnecessary antibiotic use and mitigating resistance [23,24]. However, multiplex testing can pose challenges regarding analytical sensitivity, especially critical for detecting B. pertussis, which may be present at low levels in clinical samples and can diminish quickly despite ongoing symptoms [25]. Most samples tested are often from previously vaccinated individuals, leading to atypical symptoms and a potential delay of over three weeks between symptom onset and sampling, which may result in low bacterial loads [25]. Consequently, caution is advised when interpreting negative results from the FilmArray RP2+, and supplementary diagnostic methods should be employed when necessary [26]. Other rapid diagnostic methods, such as real-time PCR (qPCR), offer improved sensitivity and can effectively differentiate between *B. pertussis* and *B. holmesii*, which can present with similar clinical manifestations [27]. While qPCR provides quick results, its reliance on specific target sequences may limit its applicability in diverse epidemiological settings. Additionally, traditional culture methods, although time-consuming, can confirm the presence of viable pathogens but may not always yield positive results if the bacterial load is low [28]. Thus, while selecting the appropriate diagnostic method, one must consider the clinical context and the likelihood of pertussis in the patient population. This is particularly important given the frequent empirical use of fluoroquinolones and β-lactams prior to diagnosis, which highlights the challenge of differentiating pertussis from other bacterial infections and the potential for inappropriate antibiotic use [29].

Hospitalization rates and complications, including severe respiratory failure and pneumonia, reflect the serious nature of pertussis in adults, especially those with comorbid conditions or advanced age. The need for intensive care in one patient highlights the severe complications that can arise and underscores the importance of early and accurate diagnosis [30,31].

The clinical outcomes in our study were generally favorable, with all patients receiving successful treatment. However, 25% reported persistent post-infectious cough at one-month follow-up, aligning with previous studies that highlight the potential for long-term respiratory complications following pertussis infection [16] and may require further management [32].

This study has several limitations that should be acknowledged. First, its observational cross-sectional design restricts the ability to establish causality between pertussis and clinical outcomes. Second, the study was conducted at a single center, which may limit the generalizability of the findings to other regions or healthcare settings with different population demographics or diagnostic practices. Additionally, although PCR testing offers high sensitivity and specificity, the lack of a gold standard comparison, such as bacterial culture, may have led to overestimation or underestimation of pertussis cases. Finally, the reliance on patient-reported vaccination histories and symptom data introduces the potential for recall bias, potentially affecting the accuracy of these variables.

## 5. Conclusions

Our study demonstrates the utility of PCR testing in improving pertussis diagnosis and emphasizes the need for enhanced vaccination strategies and ongoing surveillance. Integrating advanced diagnostic methods and updated vaccination protocols could significantly impact the management and control of pertussis, ultimately reducing its public health burden. Future research should focus on improving vaccination coverage and diagnostic accuracy to address the challenges of pertussis management effectively. Interdisciplinary collaborations between epidemiologists, clinicians, and public health officials will be essential in developing comprehensive approaches to mitigate the resurgence of pertussis and address the complexities of its epidemiology.

## Figures and Tables

**Table 1:** Demographic and clinical characteristics of patients with respiratory symptoms included in the study.

Parameters	Sum (*n* = 532)	Females (*n* = 354)	Males (*n* = 178)	*p*-Value
Age, *years (SD)*	61.87 (±13.4)	58.41 (±10.2)	65.46 (±17.4)	0.422
Pertussis, *yes,* *n (% of patients tested)*	47 (8.8)	27 (5.1)	20 (3.8)	0.831
Gender *n (% of positive cases)*	-	27 (57.4)	20 (42.6)	0.080
Known contact, *n (% of positive cases)*	6 (12.8)	4 (8.5)	2 (4.3)	0.451
Vaccination history *n (% of positive cases)*	17 (36.2)	7 (14.9)	10 (21.7)	0.347
Last dose, *years, (SD)*	46 (±14.5)	58.41 (±11.3)	35.46 (±16.4)	0.838
Symptom duration, *days, (SD)*	4.8 (±1.9)	5.4 (±1.7)	3.7 (±2.4)	0.203
Cough *n (% of positive cases)*	47 (100)	27 (57.4)	20 (42.6)	0.520
Low-grade fever *n (% of positive cases)*	17 (36.2)	12 (25.5)	5 (10.6)	0.695
Paroxysmal cough *n (% of positive cases)*	16 (34)	10 (21.3)	6 (12.8)	0.086
Wheezing *n (% of positive cases)*	8 (17)	5 (10.6)	3 (6.4)	0.079
Rhinorrhea *n (% of positive cases)*	4 (8.5)	3 (6.4)	1 (2.1)	0.075
Sneezing *n (% of positive cases)*	4 (8.5)	3 (6.4)	1 (2.1)	0.075
Headache *n (% of positive cases)*	6 (12.8)	3 (6.4)	3 (6.4)	0.581
Dyspnea *n (% of positive cases)*	6 (12.8)	2 (4.3)	4 (8.5)	0.706
Sore throat *n (% of positive cases)*	10 (21.3)	6 (12.8)	4 (8.5)	0.086
Hospitalization *n (% of positive cases)*	6 (12.8)	2 (4.3)	4 (8.5)	0.695
Complications *n (% of positive cases)*	2 (4.3)	1 (2.1)	1 (2.1)	0.203
Antibiotics prior to diagnosis *n (% of positive cases)*	44 (93.6)	27 (57.4)	20 (36.2)	0.074
Post-infectious cough *n (% of positive cases)*	11 (23.4)	6 (12.8)	5 (10.6)	0.095

## Data Availability

The original contributions presented in this study are included in the article. Further inquiries can be directed to the corresponding author.
